# Data on metabolic profiling of healthy human subjects’ plasma before and after administration of the Japanese Kampo medicine maoto

**DOI:** 10.1016/j.dib.2018.11.116

**Published:** 2018-12-03

**Authors:** Hiroyuki Kitagawa, Katsuya Ohbuchi, Masaya Munekage, Kazune Fujisawa, Yasuhiro Kawanishi, Tsutomu Namikawa, Hirotaka Kushida, Takashi Matsumoto, Chika Shimobori, Akinori Nishi, Chiharu Sadakane, Junko Watanabe, Masahiro Yamamoto, Kazuhiro Hanazaki

**Affiliations:** a Department of Surgery, Kochi Medical School, Kochi University, Kochi, Japan; b Tsumura Kampo Research Laboratories, Tsumura & CO., Ibaraki, Japan

## Abstract

This data article contains the data on metabolic profiling of healthy human subjects’ plasma before and after administration of the Japanese Kampo medicine maoto. Four healthy human subjects were recruited. Plasma samples were collected before and 0.25, 0.5, 1, 2, 4 and 8 h after maoto treatment. Endogenous and exogenous compounds in plasma were analyzed using MS. Endogenous compounds including saccharides, amino acids, organic acids and other hydrophilic metabolites were semi-quantitatively measured using GC-MS/MS. Lipid mediators such as arachidonic acid, docosahexaenoic acid and eicosapentaenoic acid were semi-quantitatively measured using LC-MS/MS. Maoto constituents in plasma were quantitatively measured using LC-MS/MS. The data files contain the area ratio values, which were normalized to the intensity of the internal standard, and plasma concentration of maoto compounds. The data article is related to the research article titled “Phenotyping analysis of the Japanese Kampo medicine maoto in healthy human subjects using wide-targeted plasma metabolomics” (Kitagawa et al., 2018).

**Specifications table**

**Table:** 

Subject area	*Biology*
More specific subject area	*Metabolomics, Traditional herbal medicine*
Type of data	*Table (Area ratio values, concentrations)*
How data were acquired	*GC-MS/MS for hydrophilic metabolites: Shimadzu GCMS-TQ8040*
	*LC-MS/MS for lipid mediators: Shimadzu LCMS-8050 with Shimadzu Nexera UPLC*
	*LC-MS/MS for maoto compounds: AB SCIEX TripleQuad6500 with Agilent 1290*
Data format	*Processed data*
Experimental factors	*Plasma samples from 4 healthy human subjects before and after administration of maoto*
Experimental features	*Wide-targeted metabolomics (lipid mediators and hydrophilic metabolites such as saccharides, amino acids and organic acids) and quantitative analysis of maoto compounds were performed.*
Data source location	*Plasma samples were collected at Kochi Medical School, Kochi University, Kochi, Japan. The analysis was performed in Tsumura Kampo Research Laboratories, Tsumura & CO., Ibaraki, Japan.*
Data accessibility	*Data are within this article*
Related research article	*Phenotyping analysis of the Japanese Kampo medicine maoto in healthy human subjects using wide-targeted plasma metabolomics*[1]

**Value of the data**

• The data demonstrate the influence of maoto administration on various plasma metabolites including maoto compounds.
• The data could be used to investigate the relationship between maoto compounds and endogenous metabolites.
• The data will be useful for other researchers to investigate herbal medicines.

## 1 Data

The data article contains the data from plasma metabolome analysis of saccharides, amino acids, organic acids, lipid mediators (Table S1), and maoto compounds (Table S2) measured using GC-MS/MS and LC-MS/MS. Data are presented with the area ratio values normalized to relevant internal standards and concentrations calculated using standard curves.

## 2 Experimental design, materials and methods

The trial was conducted at the Kochi Medical School in September 2016. The study protocol was described in [1] and approved by the Ethical Committee of Kochi Medical School. The trial was conducted in accordance with ethical norms prescribed in the Declaration of Helsinki and good clinical practice guidelines. All subjects read and signed the informed consent form prior to entering the study. The trial was registered in the University Hospital Medical Information Network (UMIN000023609; http://www.umin.ac.jp/). Four male subjects (age, 22–29 years; height, 170.5–176.5 cm; weight, 53.8–73.9 kg; and BMI, 18.5–23.7 kg/m^2^) were enrolled this study. Maoto was given orally at a dose of 7.5 g, which is the daily dosage used in clinical practice. Blood samples were collected at 0 (pre-administration), 0.25, 0.5, 1, 2, 4, and 8 h after administration of maoto (in Ref. [1, Fig. 1a]) and then, plasma samples were prepared and stored at −80 °C or below prior to GC-MS/MS and LC-MS/MS analyses.

### 2.1 Targeted metabolomics using GC-MS/MS

Plasma metabolome analysis using GC-MS/MS was performed according to a method described by Uji et al. [2]. Briefly, A 50 μL aliquot of plasma was mixed with 260 μL of a solvent mixture (MeOH:H_2_O:CHCl_3_ = 2.5:1:1, v/v/v) containing 10 μL of 0.5 mg/mL 2-isopropylmalic acid (Sigma-Aldrich) After mixing and being centrifuged supernatant (150 μL) was transferred to a clean tube and 140 μL of distilled water was added. After mixing and centrifuged, a 180 μL aliquot of the supernatant was transferred to a clean tube and lyophilized. For oximation, 80 μL of 20 mg/mL methoxyamine hydrochloride (Sigma-Aldrich) dissolved in pyridine was mixed with lyophilized sample. Samples were then shaken for 90 min at 30 °C. Then, 40 μL of *N*-methyl-*N*-trimethylsilyl-trifluoroacetamide (MSTFA) (GL Science, Tokyo, Japan) was added for derivatization and the mixture was incubated with shaking for 30 min at 37 °C. The mixture was then centrifuged, and the supernatant was subjected to measurement by GC-MS/MS.

GC-MS/MS analysis was performed using a GCMS-TQ8040 (Shimadzu, Kyoto, Japan) with a fused silica capillary column (DB-5: inner diameter, 30 m × 0.25 μm; film thickness, 1 µm; Agilent Technologies, Santa Clara, CA).

The SRM transition and average retention time of the detected metabolites were shown in Supplementary Table 1. The peak intensity of each quantified ion was calculated and normalized to that of 2-isopropylmalic acid, which was used as an internal standard.

### 2.2 Targeted metabolomics using LC-MS/MS

Plasma metabolome analysis using LC-MS/MS was performed according to a method described by Uji et al. [2]. A 1 mL aliquot of methanol with internal standard mixture was added to 200 μL of plasma sample. The mixture was mixed and centrifuged. The supernatant was diluted with 4 mL of 0.1% formic acid in water and then loaded onto a preconditioned solid-phase extraction cartridge (STRATA-X, 10 mg/1 mL, Phenomenex, Torrance, CA). The cartridge was washed, and lipids were eluted with 250 μL of 0.1% formic acid in methanol, and the eluent was evaporated and dissolved in 20 μL of methanol. A 5 μL aliquot of the sample was injected for analysis.

The LC-MS/MS system consisted of 2 LC-30AD pumps, a SIL-30AC auto-sampler, a CTO-20A column oven, a CBM-20A system controller, and a triple quadrupole mass spectrometer LCMS-8050 (Shimadzu).

Chromatogram acquisition was summarized in Table 1.

**Table 1:** LC-MS/MS metabolome analysis methods: UPLC condition.

UPLC condition
Column: Kinetex C8 (150 × 2.1 mm I.D., 2.6 μm particle size, Phenomenex, Torrance, CA)
Mobile phase (A) 0.1 vol % formic acid, (B) acetonitrile
Gradient elution program (% B in A):
0–5 min, 10–25%; 5–10 min, 25–35%; 10–20 min, 35–75%; 20–20.1 min, 75–95%; 20.1–25 min, 95%; 25–25.1 min, 95–10%.
Other conditions were: flow rate, 0.4 mL/min; column temperature, 40 °C

The SRM transition and average retention time of the detected metabolites were presented in Supplementary Table 2. The peak areas of each quantified ion were calculated and normalized to those of an internal standard mixture described in [2].

### 2.3 Quantitative analysis of the major components of maoto

Fifteen compounds were analyzed by SRM using LC-MS/MS. The plasma samples were pretreated according to the following methods prior to injection into the LC-MS/MS system.

For quantification of l-ephedrine, d-pseudoephedrine, l-methylephedrine, dl-norephedrine, and methylephedrine N-oxide, 100 µL plasma samples were pretreated by liquid–liquid extraction using ethyl acetate. The total supernatant was collected, dried, and concentrated using a centrifugal evaporator. The dried residue was then dissolved in 60 μL of the specific HPLC mobile phase used for each analytical method, and a 10 μL aliquot was injected into the LC-MS/MS system.

For quantification of amygdalin, prunasin, (E)-cinnamic acid, 2-methoxycinnamic acid, liquiritin, liquiritigenin, isoliquiritin, isoliquiritigenin, glycyrrhizic acid, and glycyrrhetinic acid, 100 µL plasma samples were pretreated by deproteinization using methanol. The supernatant was collected, dried, and concentrated using a centrifugal evaporator. The dried residue was then dissolved in 60–80 μL of the specific HPLC mobile phase used for the analytical method, and a 10 μL aliquot was injected into the LC-MS/MS system.

The LC/MS system consisted of a TripleQuad6500 (AB SCIEX, Tokyo, Japan) equipped with an Agilent 1290 system (Agilent Technologies). Analytical conditions are summarized in Tables 2 and 3.

**Table 2:** Methods of LC-MS/MS: Ion parameters of test compounds.

Compound	Q1	Q3	DP	CE	CXP	Method ID^a^
	*m/z*	*m/z*	volts	volts	volts	
*l*-ephedrin	166.152	133.1	26	29	12	1
*d*-pseudoephedrine	166.15	133.1	21	29	14	1
*l*-methylephedrine	180.149	162.1	36	19	14	1
*dl*-norephedrine	151.971	134.1	6	15	12	1
Methylephedrine N-oxide	196.098	62.1	31	19	8	1
Amygdalin	456.082	323.1	−155	−18	−15	2
Prunasin	293.964	161	−110	−12	−25	2
(*E*)-cinnamic acid	146.911	103	−70	−14	−3	2
(*E*)-2-methoxycinnamic acid	177.001	132.9	−25	−16	−15	2
Liquiritin	417.164	254.9	−75	−26	−23	2
Liquiritigenin	255.037	119	−65	−30	−11	2
Isoliquiritin	417.084	254.9	−150	−26	−27	2
Isoliquiritigenin	254.978	118.8	−20	−30	−15	2
Glycyrrhetinic acid	471.234	91	11	111	16	3
Glycyrrhizic acid	840.37	453.3	31	43	8	3
Atropine (IS)	290.019	124.1	111	31	14	1, 3
Niflumic acid (IS)	280.826	236.8	−60	−30	−16	2

a : Method ID is linked to number described in Supplementary Table 2. We used a LC-MS/MS system in this study as follows: a TripleQuad6500 (AB SCIEX, Tokyo, Japan) equipped with an Agilent 1290 system (Agilent Technologies, Tokyo, Japan). DP, declustering potential; CE, collision energy; CXP, collision cell exit potential.

**Table 3:** LC-MS/MS methods: HPLC condition.

Method ID	HPLC condition
1	Column: Inertsil Ph-3 column (100 × 2.1 mm I.D., 3 μm particle size; GL Sciences, Tokyo, Japan)
	Guard column: Opti-guard C18 (φ 1 mm × 15 mm; Optimize Technologies, Oregon City, OR)
	Mobile phase (A) 0.2 vol % formic acid, (B) acetonitrile
	Gradient elution program (% B in A):
	0–9 min, 2%; 9–15 min, 2–90%; 15–15.01 min, 90–2%; 15.01–20 min, 2%
	Other conditions were: flow rate, 0.4 mL/min; column temperature, 40 °C
2	Column: Ascentis Express RP-amide column (100 × 2.1 mm I.D., 2.7 μm particle size; Supelco, Bellefonte, PA)
	Guard column: Ascentis Express RP-Amide guard cartridge (5 × 2.1 mm I.D., 2.7 μm particle size; Supelco)
	Mobile phase (A) 0.2% vol acetic acid, (B) acetonitrile containing 0.2% vol acetic acid
	Gradient elution program (% B in A):
	0–5 min, 7%; 5–20 min, 7–95%; 20–20.01 min, 95–7%; 20.01–25 min, 7%
	Other conditions were: flow rate, 0.35 mL/min; column temperature, 40 °C
3	Column: CAPCELL CORE ADME (100 × 2.1 mm I.D., 2.7 μm particle size; Shiseido, Tokyo, Japan)
	Guard column: CORE ADME EXP cartridge (5 × 2.1 mm I.D., 2.7 μm particle size; Shiseido)
	Mobile phase (A) 10 mmol/L ammonium acetate, (B) methanol
	Gradient elution program (% B in A):
	0–1 min, 30%; 1–3 min, 30–50%; 3–10 min, 50–95%; 10–15 min, 95%; 15–15.01 min, 95–30%; 15.01–20 min, 30%
	Other conditions were: flow rate, 0.3 mL/min; column temperature, 40 °C

As an internal standard, niflumic acid (Sigma-Aldrich) and atropine sulfate monohydrate (Wako Pure Chemicals Industries) were selected based on consideration of the characteristic of the analytes, as these compounds were not detected in control plasma and did not overlap with other targets with respect to retention time and SRM transition.

### 2.4 Data processing

Processing of metabolomics data was performed using Microsoft Excel software. Missing values in the raw data were replaced by half of the minimum positive value for each metabolite. In addition, metabolites with normalized area variation in pooled plasma samples >30% were removed from the detected metabolites since they were unstable [3].

The concentration of major components of maoto at each timepoint was calculated as the mean value, but the mean value was designated as zero when the concentration was less than half of the quantification limit.
