# Supplementary material for: Data on metabolic profiling of healthy human subjects’ plasma before and after administration of the Japanese Kampo medicine maoto
Source: Data Brief. 2018 Dec 3;22:359–64. doi: 10.1016/j.dib.2018.11.116 (PMC6307690; doi:10.1016/j.dib.2018.11.116)
Supplement: Supplementary file 1 — Supplementary material [file mmc1.zip › Conflict of interest form_2.pdf]

Signature: Kiyohiro Haruyuki  Date: 2018/11/14

Signature: Toritomu Namikawa  Date: Nov. 14, 2018

Signature: Hiroyuki Kitagawa  Date: Nov. 14, 2018

Signature: Masaya Munekage  Date: Nov. 15, 2018

Signature: kosyune fujisawa  Date: Nov. 15. 2018

Signature: Yasuhiro Kawanishi  Date: NOV. 15. 2018.

Signature:  Date:

Signature:  Date:

Signature:  Date:

Signature:  Date:

Signature:  Date:

Signature:  Date:

Signature:  Date:

Signature:  Date:
