# Supplementary material for: Data on metabolic profiling of healthy human subjects’ plasma before and after administration of the Japanese Kampo medicine maoto
Source: Data Brief. 2018 Dec 3;22:359–64. doi: 10.1016/j.dib.2018.11.116 (PMC6307690; doi:10.1016/j.dib.2018.11.116)
Supplement: Supplementary file 1 — Supplementary material [file mmc1.zip › Conflict of interest form_1.pdf]

Conflict of interest form

We wish to draw the attention of the Editor to the following facts which may be considered as potential conflicts of interest and to significant financial contributions to this work.
Katsuya Ohbuchi, Hirotaka Kushida, Takashi Matsumoto, Chika Shimobori, Akinori Nishi, Chiharu Sadakane, Junko Watanabe and Masahiro Yamamoto are employees of Tsumura & Co. Hiroyuki Kitagawa, Masaya Munekage, Kazune Fujisawa, Yasuhiro Kawanishi, Tsutomu Namikawa and Kazuhiro Hanazaki have no conflict of interest.

We confirm that the manuscript has been read and approved by all named authors and that there are no other persons who satisfied the criteria for authorship but are not listed. We further confirm that the order of authors listed in the manuscript has been approved by all of us.

We confirm that we have given due consideration to the protection of intellectual property associated with this work and that there are no impediments to publication, including the timing of publication, with respect to intellectual property. In so doing we confirm that we have followed the regulations of our institutions concerning intellectual property.

We further confirm that any aspect of the work covered in this manuscript that has involved human patients has been conducted with the ethical approval of all relevant bodies and that such approvals are acknowledged within the manuscript.

We understand that the Corresponding Author is the sole contact for the Editorial process (including Editorial Manager and direct communications with the office). He is responsible for communicating with the other authors about progress, submissions of revisions and final approval of proofs. We confirm that we have provided a current, correct email address which is accessible by the Corresponding Author and which has been configured to accept email from oobuchi_katsuya@mail.tsumura.co.jp

Signed by all authors as follows:

Signature: Katsuya Ohbuchi Date: 11/13/2018.

Signature: Takashi Matsumoto Date: 11/13/2018

Signature: Chiharu Sadakane Date: 11/13/2018

Signature: Hirotaka Kushida Date: 11/13/2018

Signature: Akinori Nishi Date: 11/13/2018

Signature: Chika Shimobori Date: 11/14/2018

Signature: Junko Watanabe Date: 11/14/2018

Signature: Masahiro Yamamoto Date: 11/14/2018

Signature: Date:

Signature: Date:

Signature: Date:

Signature: Date:

Signature: Date:

Signature: Date:
